# Vomiting Connected with Anæsthesia
^1^Read before the Bristol Medico-Chirurgical Society on December 9th, 1908.


**Published:** 1909-03

**Authors:** A. L. Flemming

**Affiliations:** Hon. Anæsthetist to the Bristol Royal Infirmary


### VOMITING CONNECTED WITH ANÆSTHESIA.[1]

BY

A. L. Flemming, M.R.C.S., L.R.C.P.,
*Hon. Anæsthetist to the Bristol Royal Infirmary.*

In an endeavour, which has recently been made at the Royal Infirmary, to reduce the frequency and to diminish the severity of vomiting associated with general anæsthesia, the methods adopted of preparing the patients and of administering the drugs have, we think, produced results, sufficiently definite and encouraging, to justify our bringing the matter before the notice of this Society, in the hope that members may give us the benefit of their experience, especially as regards post-anæsthetic vomiting, and may thus help us to deal with those cases, namely 37 per cent. of all, in whom, at present, sickness seems to be unavoidable.

Our series of cases consists of 500 patients, operated on in the two main theatres, taken consecutively. And notice is taken of all vomiting, including the ejection of mucus, which occurred during the twenty-four hours following the induction of anæsthesia.

The results were as follows :—

| | Vomited. |
|---|---|
| In all .. .. .. .. .. .. | 37 per cent. |
| After consciousness returned .. .. | 5 per cent. |
| After twelve hours from end of operation .. | 2½ per cent. |

[1] Read before the Bristol Medico-Chirurgical Society on December 9th, 1908.

Besides being a source of suffering to the patient and of annoyance to the surgeon, vomiting may lead to very serious complications, such as syncope or pulmonary affections.

The circulatory depression which precedes emesis is generally transient, and disappears when the act of vomiting is over. But in certain cases, especially in those recovering from chloroform anæsthesia, this faintness may prove, and has proved, fatal; a result which is not to be wondered at when one realises that, for the time being, the patient shows many of the cardinal signs of shock, namely pallor, cold and clammy condition of skin, shallow respiration, rapid and extremely soft pulse, and sometimes insensitive cornea or dilated pupils; the order of events which culminate in the fatal result being probably (1) depressed respiration or—if retching occur—closure of the glottis, (2) consequent imprisonment in the body of chloroform vapour and carbon dioxide, and a coincident deprivation of oxygen; these factors, of course, constituting a relative overdose of chloroform.

A similar train of incidents may occur at any moment when the anæsthesia is unduly light, and probably bear an important causal relationship to the occurrence of many deaths in the induction stage of chloroform anæsthesia, 50 per cent. of the deaths which occur under chloroform taking place in this stage.

The tendency to vomit, especially when the patient is but lightly "under" at the commencement or end of anæsthesia, is greatly increased by any movement of the trunk—as from one table to another, or as in rolling over for bandaging.

It is my belief that post-anæsthetic bronchitis and pneumonia are caused by the inhalation of vomited matter more often than is generally supposed. One marked illustration of this occurred in my hands some months ago at the Infirmary, when a healthy man, aged 22, vomited mucus while being rolled over for bandaging after operation for hernia. The act of vomiting was extremely sudden and forcible, and the following inspiration was heard to draw fluid into the windpipe. The stethoscope immediately revealed copious bubbling in the left chest, and twenty-four hours later the patient had developed left-sided broncho-pneumonia.

Measures by which vomiting and its complications may be to some extent obviated include (1) preparation of patient, (2) method of administering the drugs, (3) management of patient during anæsthesia, (4) management after operation.

(1) *Management of patient.*—In our series the method of preparation differed from the course usually adopted in the substitution of boiled or peptonised milk for the time-honoured beef-tea. The patients were in most instances allowed ordinary mixed, but plain, diet on the day preceding, but on the day of operation they had bread and butter and tea at 6.30 a.m., and at 9.30 from two to four ounces of milk, the operating hour being 1.0 p.m. In special cases milk diet was given during the day preceding operation. Repeated control experiments pointed to the desirability of omitting the usual beef-tea.

(2) *Administration.*—Where permissible anæsthesia was induced by gas or ethyl-chloride and ether, and was maintained by ether for the first twenty or thirty minutes, after which time as a rule chloroform and ether were given on a domette Murray's mask by the two-bottle method, the fabric being kept moist with ether from one bottle and the chloroform being added from the other bottle in very small quantities, one drop in every four, five, six, or even seven breaths being sufficient to maintain an even anæsthesia. The total quantity of chloroform used being often as small as four or five drams per hour. This two-bottle system has many advantages, and has been used by some administrators at the Infirmary since 1895 ; and in this connection it is interesting to note that Tyrrell, in introducing his two-bottle Junker apparatus in 1897, claimed as one of its advantages a diminution in the frequency of vomiting.

*Management during anæsthesia.*—Unfortunately our patients had to be lifted from the anæsthetic to the operating table, and thence to the stretcher, and finally from the stretcher to the bed, and we feel that much might be done to eliminate some of this movement and jolting.

*Management after operation.*—To avoid vomit being inhaled, and to prevent cyanosis from tongue obstruction, every patient should (where permissible) be kept on his side until fully conscious.

There still seems to be a regrettable deep-seated tendency among nurses to regard the supine as the normal post-operation posture.

Mr. S. V. STOCK said that he could fully endorse the value of Mr. Flemming's methods, particularly the substitution of milk-feeds for beef-tea before operation. Prolonged fasting was bad. A sister at a children's ward had remarked to him that occasionally children preparing for operation refused to take the milk-feed during the morning before operation, and such children nearly always vomited ; only a small proportion vomited of those who took the milk. Lavage of the stomach would not always prevent vomiting if the operation was protracted. He would always wash out the stomach before, rather than after, the patient was anæsthetised.

Mr. HEY GROVES said that movement of the patient could not always be avoided ; the "fear of the table" made it necessary in some cases to administer the anæsthetic elsewhere than on the operating table. After the operation patients do not vomit until the anæsthetic is stopped ; therefore he advised keeping them fully anæsthetised until the bandaging was finished and the patient returned to bed. He had a high opinion of adrenalin solution in treating post-operative hæmatemesis.

Mr. PAUL BUSH suggested that the patient should be anæsthetised in an adjoining room upon the actual operating table, which could then be wheeled into the theatre.

Dr. SYMES maintained that the duration of the operation influenced the frequency of vomiting, which was the result not only of the drug administered but also of the nervous shock. This shock was proportional in some measure to the length of the operation ; moreover, nervous people often experienced nausea and even vomiting after administration of nitrous oxide. Two drugs, morphia and atropine, administered in water before the operation appeared each to lessen vomiting.

Dr. BERTRAM ROGERS inquired whether the various commercial methods of preparing chloroform or ether could be said to affect vomiting.

Dr. OGILVY drew attention to the proneness of ophthalmic cases to vomit during the course of operation.

Dr. FORTESCUE-BRICKDALE remarked that beef-tea stimulated gastric secretion, and would on that account be more likely to contribute to vomiting than milk.

Mr. LACE quoted the experience of a colleague who had found that the administration of nothing but peptonised milk for twenty-four hours before operation diminished the chances of anæsthetic vomiting, and on inquiry he had found a consensus of opinion among nurses to the same effect.

Mr. FLEMMING in reply said that preliminary lavage of the stomach sometimes gave the anæsthetist a false sense of security. In fæcal vomiting it was useless, for during a long operation regurgitation into the stomach provided fresh material to be vomited. The suggestion of anæsthetising patients on the operating table would only affect the vomiting during induction of anæsthesia. He objected to the preliminary use of morphia or atropine on account of the difficulties they introduced during anæsthesia. As to the use of acetone chloroform, he thought that with the addition of sufficient ethyl chloride to render the evaporation point stable it was a safe anæsthetic, but he preferred to administer a preparation about which there was no doubt.

